# Use of olanzapine compared with clozapine for treatment-resistant schizophrenia in a real-world setting: nationwide register-based study

**DOI:** 10.1192/bjo.2021.964

**Published:** 2021-08-03

**Authors:** Young Tak Jo, Sung Woo Joo, Soojin Ahn, Youngjae Choi, Jungsun Lee

**Affiliations:** Department of Psychiatry, Asan Medical Center, University of Ulsan College of Medicine, Republic of Korea; Department of Psychiatry, Asan Medical Center, University of Ulsan College of Medicine, Republic of Korea; Department of Psychiatry, Asan Medical Center, University of Ulsan College of Medicine, Republic of Korea; Department of Psychiatry, Asan Medical Center, University of Ulsan College of Medicine, Republic of Korea; Department of Psychiatry, Asan Medical Center, University of Ulsan College of Medicine, Republic of Korea

**Keywords:** Schizophrenia, clozapine, olanzapine, national health programs, delivery of healthcare

## Abstract

**Background:**

Clozapine is generally considered as the treatment of choice for patients with treatment-resistant schizophrenia (TRS). However, its superiority has recently been questioned because olanzapine has been suggested as non-inferior to clozapine in its effectiveness.

**Aims:**

We aimed to investigate the current status of clozapine prescriptions to identify any disparity between clinical guidelines and real-world practices.

**Method:**

In this study, we utilised the Health Insurance Review Agency database in the Republic of Korea to investigate the real-world effectiveness of clozapine for patients with TRS. We compared differences in patient variables before and after clozapine administration, and we also performed survival analyses for both psychiatric admissions and emergency room visits among patients who used clozapine or olanzapine.

**Results:**

This study investigated an incident cohort of 64 442 patients, and 2338 patients have been prescribed clozapine. Of these, 998 patients had TRS. In survival analysis, clozapine showed a worse survival rate for psychiatric admissions than olanzapine (hazard ratio 0.615). We also identified that clinicians tended to try a number of antipsychotics, as recommended, before starting patients on clozapine.

**Conclusions:**

In conclusion, we found that olanzapine led to higher survival rates for psychiatric admissions than clozapine. Thus, considering the risk of serious adverse effects, clozapine may be used conservatively. Considering several studies advocating superior efficacy of clozapine, further studies with extensive data are recommended.

## Background

Schizophrenia is a chronic and debilitating disorder characterised by hallucinations, delusions and disorganised thoughts. As schizophrenia reduces the mental function of patients and often seriously impairs their quality of life, aggressive treatment is often considered. However, approximately 30% of patients with schizophrenia cannot achieve remission, even with appropriate antipsychotics use.^1^ This is referred to as treatment-resistant schizophrenia (TRS).^2^ According to the available clinical guidelines,^3,4^ clozapine is generally considered as the treatment of choice for patients with TRS.

## Clozapine

Clozapine, which was first sold commercially in 1972 as the first atypical antipsychotic, has been known as the most effective antipsychotic. despite some adverse effects including fatal agranulocytosis.^5^ It has been widely prescribed because of its higher efficacy compared with other atypical antipsychotics. Several studies have demonstrated the efficacy and cost-effectiveness of clozapine for patients with TRS.^6–9^

## Olanzapine

However, the superiority of clozapine has recently been questioned. Some studies have suggested that the superiority of clozapine compared with olanzapine in terms of effectiveness is uncertain.^10–13^ In other words, olanzapine is non-inferior to clozapine. Olanzapine has been considered superior to other atypical antipsychotics.^14^ Based on this perspective, considering the risk of serious adverse effects^15^ and the cost of routine laboratory check-ups, more conservative usage of clozapine might be considered.

## Aims

A comprehensive study using real-world data is therefore warranted. It should be clarified whether or not clozapine should be used as the treatment of choice for patients with TRS. It has been suggested that real-world practice relating to antipsychotics may be different from well-designed study settings.^16^ Although a few studies have investigated real-world data for clozapine use,^16–18^ this has been limited considering the widespread use of clozapine. It has been challenging to investigate real-world data for patients who use clozapine because most countries do not have a nationwide healthcare register for such research.

In this study, we utilised the Korean nationwide healthcare register to investigate the real-world effectiveness of clozapine for patients with TRS. Specifically, we investigated data from the Health Insurance Review & Assessment Service (HIRA) database between 1 January 2007 and 31 December 2016, to determine the real-world effectiveness of clozapine for patients with TRS, especially compared with olanzapine, and we also identified additional information including the prevalence of use and prescribing patterns.

## Method

### Nationwide register (HIRA)

In this study, we utilised the data from the HIRA database.^19^ In the Republic of Korea, there is a single government-payer health coverage system, the National Health Insurance Service, that covers approximately 98% of the national population. The HIRA can be used to review medical fees and the attributes of healthcare. For this purpose, HIRA has constructed and maintained a vast nationwide database comprising all medical claims. Since the HIRA database covers nearly all of the nation's population, this database advantageously represents the entire Korean population, making it a valuable source for public health research.

### Study population and TRS

This study was approved by the Institutional Review Board of Asan Medical Center (File Number: 2018–0131) and the requirement for informed consent from patients were exempted because the HIRA database consists of de-identified data.

We extracted the claim data from 1 January 2007 to 31 December 2016 (Serial number: M20180205894) from the HIRA database. This data included the following patient information: age, gender, date of admission, number of days of hospital admission, psychiatric diagnosis, prescribed medications. We then extracted data for patients who had at least one claim for the Korean Standard Classification of Diseases (KCD)^20^ diagnostic code of F20 (schizophrenia) during the study period. As a reference, the KCD is based on the ICD-10.^21^

Using the data extracted for patients with schizophrenia, we defined an incident cohort to represent real-world situations more accurately. The incident cohort was defined as patients diagnosed with F20 for the first time between 2007 and 2016. The included patients had to have been antipsychotic-free for at least 1 year preceding the index point, to completely remove returning patients from the data-set, and patients had to have received antipsychotic treatment within 3 days of diagnosis. Patients with intellectual disability, pervasive developmental disorder and organic brain syndrome, for whom the schizophrenia diagnostic code is often used because of their psychotic symptoms, were also excluded. In addition, we further excluded patients who were under 12 or over 80 years of age at the time of diagnosis to maintain homogeneity of the included patients.

The cohort entry point was defined as the day when each patient was diagnosed with schizophrenia, and the follow-up time was from diagnosis to the end of the study period or death. TRS was defined as schizophrenia in which physicians prescribed more than two kinds of different antipsychotics for treatment. These antipsychotics have to be administered for at least 6 weeks and should reach the optimal therapeutic dosage of 10 mg olanzapine or equivalent, after titration. Because this was a register-based study, we allowed some periods with polypharmacy.

### Analysis of before and after clozapine administration

Several variables related to clinical courses were investigated and compared from before and after the use of clozapine. In particular, we compared the numbers of psychiatric admissions and emergency room visits, and the medication possession ratio (MPR). MPR, one of the most common measures of medication adherence, was calculated as the percentage of days of supply received divided by a period of time.

### Clozapine versus olanzapine

We performed survival analysis to investigate the real-world effectiveness of clozapine longitudinally. To determine whether or not clozapine is superior in terms of real-world effectiveness, we compared clozapine with olanzapine for patients with TRS. As it is common to take multiple antipsychotics concurrently in many of these patients (pure monotherapy is relatively rare), we compared patients who used clozapine plus other antipsychotics other than olanzapine, versus patients who use olanzapine plus other antipsychotics other than clozapine in survival analysis. In addition, we also compared base demographics and clinical characteristics between these groups to further explain the differences in survival rates.

For survival analyses, both psychiatric admissions and emergency room visits were defined as the primary outcomes. The Kaplan–Meier survival curves were drawn for each survival analysis. To calculate hazard ratios for admissions and emergency room visits for clozapine compared with olanzapine, the Cox proportional hazards model as applied.

For sensitivity analysis, the age at disease onset and the number of antipsychotics used before clozapine or olanzapine were considered covariates to minimise selection bias.

### Current status of clozapine prescription

Lastly, we investigated the current status of clozapine prescriptions. We estimated the mean treatment time before the initial use of clozapine, and also how many kinds of antipsychotics were used before clozapine. In so doing, we investigated the disparity between clinical guidelines and real-world practices. For the exclusion of unusually short or inappropriate administrations of antipsychotics, we set similar parameters as for investigating admissions. The antipsychotics that were administered for over 6 weeks and reached optimal therapeutic dosage after titration, as described in the Study population and TRS section above, were investigated.

### Statistical analyses

We performed the paired *t*-test, Pearson chi-squared test and Kruskal–Wallis chi-squared test for the comparisons between patient groups. For survival analyses, we utilised the Cox proportional hazards model. All data were processed and analysed using R 3.4.1.^22^ Statistical significance was set at *P* < 0.05.

## Results

### Study population and demographics

The number of patients who had at least one claim for the F20 (schizophrenia) diagnostic code totalled 448 889 according to the HIRA data. This equated to 0.88% of the Korean national population, which was 51 245 707 in 2016.

The incident cohort was defined as patients diagnosed as F20 for the first time between 2007 and 2016 and consisted of 252 299 patients. Within the incident cohort, 129 341 patients were included after excluding intellectual disability, pervasive developmental disorder and organic brain syndrome. After excluding patients who were under 12 or over 80 years of age at the time of diagnosis, there were 126 755 patients remaining. In total, 64 442 patients were included in the final investigations, after excluding patients who did not receive antipsychotics within 3 days of diagnosis or who were not antipsychotic-free for 1 year preceding the index point.

**Fig. 1:**
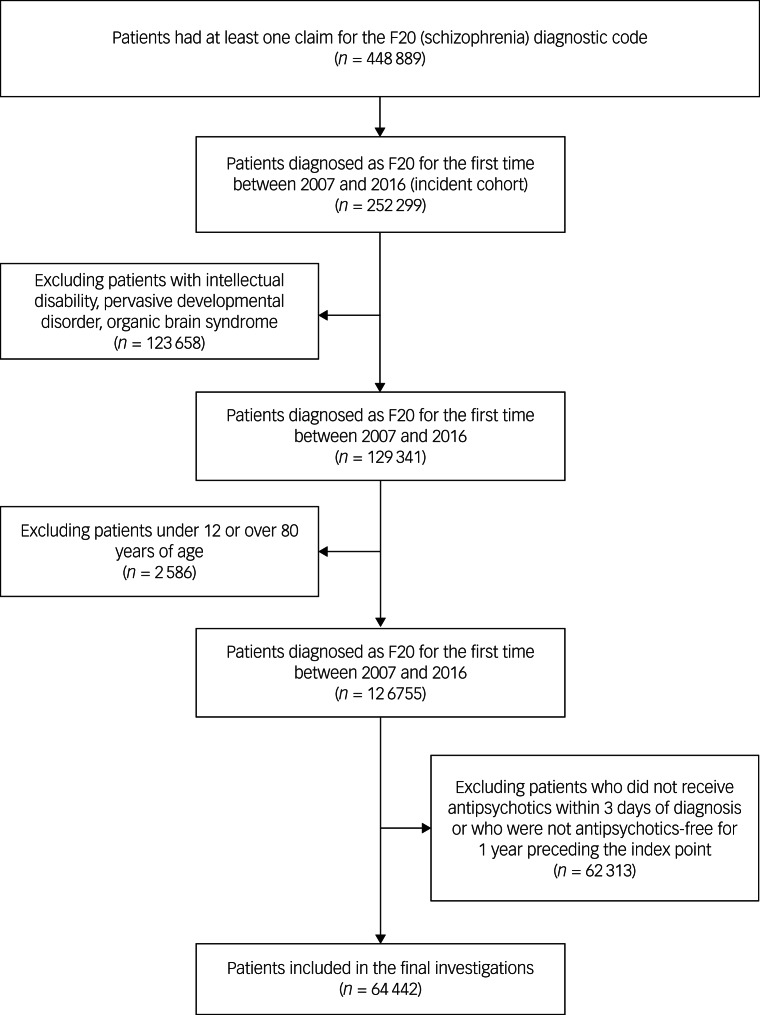
Patient eligibility and the flow of patients included in or excluded from the study.

The mean age of the incident cohort was 40.9 (s.d. = 15.6) years, and 29 335 patients were men. The patients in this cohort were treated for 1391.3 (s.d. = 1041.2) days on average. In total, 52 000 patients had experienced at least one treatment discontinuation, defined as a medication-free interval longer than 28 days. For the discontinued patients, an average of 2.29 (s.d. = 2.66) discontinuations was reported. Figure 1 summarises patient eligibility and the flow of patients included in the study.

### Analysis of before and after clozapine administration

In the incident cohort, 2338 patients have been prescribed clozapine. Of the 2338 patients who used clozapine, 998 patients were revealed as having TRS. The mean onset age was 33.5 (s.d. = 13.1) years, and 465 patients (46.6%) were men.

Among patients with TRS who used clozapine, the total number of psychiatric admissions was 3162 before clozapine introduction, and 1046 after. Moreover, the total number of emergency room visits, before and after clozapine introduction were 1067 and 254, respectively.

The total observation period before and after clozapine introduction was 1 210 842 days and 429 571 days, respectively. Thus, the psychiatric admissions per unit day before and after clozapine introduction were 0.953 and 0.889, respectively. Similarly, emergency room visits per unit day before and after clozapine introduction was 0.322 and 0.216, respectively.

The average MPR was higher after clozapine introduction (0.879 (s.d. = 0.164) *v.* 0.767 (s.d. = 0.199), *t* = −14.5, d.f. = 997, *P* < 0.001). Table 1 shows a detailed comparison between before and after clozapine administration.

**Table 1:** Comparison between before and after starting clozapine treatment

Patients with treatment-resistant schizophrenia	Before clozapine	After clozapine
Total, *n*	998	
Male gender, *n* (%)	465 (46.6)	–
Age, years: mean (s.d.)^a^	33.5 (13.1)	–
Total observation periods, days: *n*	1 210 842	429 571
Admissions, *n* (*n* per year)	3162 (0.953)	1046 (0.889)
Emergency room visits, *n* (*n* per year)	1067 (0.322)	254 (0.216)
Medication possession ratio, mean (s.d.)*	0.767 (0.199)	0.879 (0.164)

a. Age at introduction of clozapine.

* Statistically significant *P* < 0.05.

### Clozapine versus olanzapine

There were 578 patients with TRS who used clozapine (who were also olanzapine-free) and 1470 patients who used olanzapine (who were also clozapine-free). The mean onset age for patients who used clozapine was 30.5 (s.d. = 12.9) years, which was significantly younger than the 36.6 (s.d. = 14.1) years of those who used olanzapine (Kruskal–Wallis χ^2^ = 82.007, d.f. = 1, *P* < 0.001).

The total duration of treatment was longer in the clozapine group than in the olanzapine group (407.5 (s.d. = 554.9) days *v.* 313.9 (s.d. = 438.4) days, Kruskal–Wallis χ^2^ = 5.431, d.f. = 1, *P* = 0.020). Furthermore, it took an average of 1258.3 (s.d. = 785.2) days on average to start clozapine, which was not statistically significantly different from the average of 1268.9 (s.d. = 748.7) days for olanzapine (Kruskal–Wallis χ^2^ = 0.444, d.f. = 1, *P* = 0.505). Table 2 presents the demographics and clinical characteristics of the clozapine and olanzapine groups.

**Table 2:** Demographics and clinical characteristics of clozapine and olanzapine groups of patients with treatment-resistant schizophrenia

	Clozapine (*n* = 578)	Olanzapine (*n* = 1470)	χ^2^	d.f.	*P*
Male gender, *n*	259	646	0.093^a^	1	0.7604
Onset age, years: mean (s.d.)	30.5 (12.9)	36.6 (14.1)	82.007^b^	1	<0.001*
Time to start medication: mean (s.d.)	1258.3 (785.2)	1268.9 (748.7)	0.444^b^	1	0.505
Duration of treatment: mean (s.d.)	407.5 (554.9)	313.9 (438.4)	5.431^b^	1	0.020*

a. Pearson chi-squared.

b. Kruskal–Wallis chi-squared.

* Statistically significant at *P* < 0.05.

**Fig. 2:**
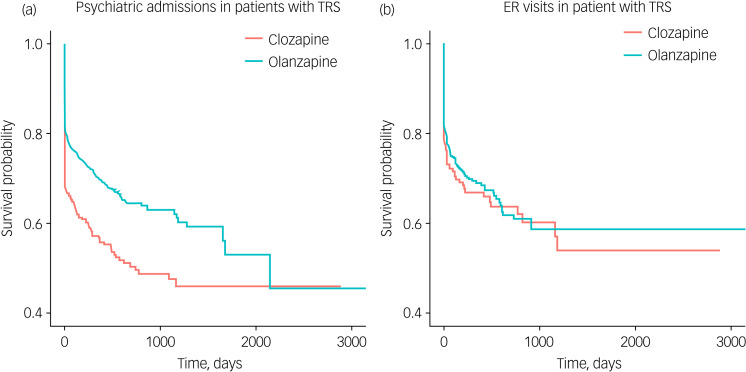
Survival curves for clozapine versus olanzapine. (a) Psychiatric admissions to hospital and (b) emergency room (ER) visits by patients with treatment-resistant schizophrenia (TRS).

In survival analysis, olanzapine showed a superior survival rate for psychiatric admissions (hazard ratio (HR) = 0.615, 95% CI 0.523–0.722, *P* < 0.001). There was no statistically significant difference in survival rates for emergency room visits between the clozapine and olanzapine groups (HR = 0.909, 95% CI 0.711–1.162, *P* = 0.447). Figure 2 shows the survival curves for clozapine versus olanzapine.

### Current status of clozapine and olanzapine use

The mean treatment time before the initial usage of clozapine was 655.0 (s.d. = 512.6) days. It was estimated that an average of 2.91 (s.d. = 1.16) kinds of antipsychotics were used before the prescription of clozapine. For olanzapine, the mean treatment time before the initial usage was 617.1 (s.d. = 524.8) days. It was estimated that an average of 2.40 (s.d. = 0.72) kinds of antipsychotics was used before the prescription of olanzapine. Table 3 show the current status of clozapine and olanzapine use.

**Table 3:** Current status of clozapine and olanzapine use in patients with schizophrenia

	Clozapine, mean (s.d.)	Olanzapine, mean (s.d.)
Total treatment time before using current medication, days	898.9 (646.1)	907.0 (629.1)
Only those with adequate antipsychotic treatment^a^	655.0 (512.6)	617.1 (524.8)
Number of different antipsychotics before using medication	5.75 (1.86)	4.49 (1.58)
Only those with adequate antipsychotic treatment^a^	2.91 (1.16)	2.40 (0.72)

a. Antipsychotics administered for more than 6 weeks and reached a dose equivalent to 10 mg olanzapine after titration (i.e. patient with treatment-resistant schizophrenia).

## Discussion

In this study, we tried to comprehensively investigate whether or not clozapine should always be used as the treatment of choice for patients with TRS. A few studies^10–13^ have suggested that clozapine may not be superior to other atypical antipsychotics such as olanzapine in terms of effectiveness. Indeed, in some cases, patients who were non-responsive to clozapine later respond to olanzapine.^23^

As a high-efficacy antipsychotic, olanzapine has been considered as superior to other atypical antipsychotics.^14,24^ Based on this perspective, and considering the risk of serious adverse effects and the cost of routine laboratory check-ups, clozapine should be used conservatively. Thus, a comprehensive study using real-world data was warranted. Herein, we utilised the HIRA data from Korea to document the real-world effectiveness and appropriateness of clozapine as the treatment of choice for TRS, taking another plausible choice of antipsychotic into consideration.

### Main findings and interpretation of our findings

We compared the clinical characteristics of patients with schizophrenia before and after starting clozapine. The numbers of psychiatric admissions and emergency room visits per year were lower in patients after starting clozapine, compared with before starting clozapine. However, due to the methodological limitations, we could not elucidate any statistical differences. We also identified that both admissions and emergency room visits rarely occurred. Furthermore, our study showed that MPR, a significant measure of treatment adherence, was higher after the introduction of clozapine. This suggests that treatment adherence for each patient may improve after the introduction of clozapine.

Despite a high MPR, clozapine did not show significant effectiveness superior to olanzapine in our primary survival analysis. In our survival analyses, the two different variables, psychiatric admissions and emergency room visits were examined and clozapine and olanzapine compared. These results were not supportive of clozapine, although the treatment adherence represented by MPR improved after clozapine initiation. In other words, clozapine did not show better clinical effectiveness despite better treatment adherence.

In the survival analysis, clozapine showed worse effectiveness in terms of admissions compared with olanzapine. As hospital admissions has been widely considered as an appropriate proxy for the clinical effectiveness of treatment,^25–27^ we could assume that olanzapine showed at least non-inferior effectiveness compared with clozapine within the patients with TRS data. In fact, some studies have already suggested that olanzapine may have at least the same effectiveness in patients with schizophrenia, especially in terms of cognitive effects, compared with clozapine.^28–30^ There were no statistically significant differences in the efficacies between clozapine and olanzapine in terms of positive or negative symptoms in a systematic review, although clozapine may have shown a little better efficacy than other antipsychotics.^31^

### Limitations

There are some limitations to this study. Because our study is a register-based study, we could not completely rule out the possibilities of undetected earlier episodes in our investigated patients. Also, we could not entirely exclude returning patients since we set a 1-year antipsychotic-free period to define the incident cohort. However, in a sensitivity analysis with a 3-year antipsychotic-free period, we found generally similar tendencies from the results. Moreover, there could be selection bias in the clozapine and olanzapine groups. As this was not a randomised controlled trial, patients who used clozapine could generally have more severe symptoms compared with patients who used olanzapine. Indeed, patients who used clozapine used more antipsychotics before the target antipsychotic (in this case, clozapine) and were also younger than those who used olanzapine in our demographic comparisons. It has been suggested that patients with early onset of schizophrenia show higher impulsivity.^32^ However, we achieved results with the same consistencies after introducing the age of disease onset and the number of antipsychotics used before clozapine or olanzapine in a sensitivity analysis. Furthermore, we tried to mitigate these limitations by restricting our focus of analysis within the patients with TRS who had already tried more than two different antipsychotics, which may have alleviate the possible differences in disease severity. Considering these limitations, we can only suggest that olanzapine is non-inferior to clozapine, with significantly better survival rates.

### Prescribing hesitancy

Finally, we aimed to reveal the current status of clozapine use in this study, especially compared with current clinical guidelines. According to several guidelines relating to first-episode psychosis, the general recommendation is to use clozapine after two trial failures of antipsychotics with optimum dosages.^3,33^ For instance, it has been recommended to ‘offer clozapine to people with schizophrenia whose illness has not responded adequately to treatment despite the sequential use of adequate doses of at least two different antipsychotic drugs’ in the UK.^3^ Contrary to the clinical guidelines, we found that clinicians generally used clozapine far more conservatively. Some patients had been administered over nine different kinds of antipsychotics before clozapine was prescribed. In other words, clinicians tended to resist prescribing clozapine in real-world situations. A few studies have already identified this hesitancy among clinicians.^34,35^ This may in part be because of clozapine's dangerous adverse effects, such as agranulocytosis. However, taking our survival analysis into consideration, it may also be because of the recognised limited effectiveness of clozapine among clinicians in the real-world.

### Implications

In conclusion, we investigated whether or not clozapine should always be considered the most effective antipsychotic in patients with TRS. We showed that olanzapine had higher survival rates than clozapine in terms of psychiatric admissions. Accordingly, it may be implied that olanzapine has at least non-inferior effectiveness compared with clozapine. This suggests that clozapine may not be the only treatment of choice for TRS. However, since many studies advocate the superior efficacy of clozapine, further longitudinal studies with extensive data spanning a few decades are recommended.

## Data Availability

The data that support the findings of this study are not publicly available because of the restrictions by the Health Insurance Review & Assessment Service.
